# The Association of Work-related Stress According to the Demand–Control Model With Aggravation of Pre-existing Disease During the First State of COVID-19 Emergency in Japan

**DOI:** 10.2188/jea.JE20210146

**Published:** 2021-12-05

**Authors:** Yupeng He, Hiroshi Yatsuya, Chifa Chiang, Atsuhiko Ota, Ryo Okubo, Tomohiro Ishimaru, Takahiro Tabuchi

**Affiliations:** 1 Department of Public Health and Health Systems, Nagoya University Graduate School of Medicine, Aichi, Japan; 2 Department of Public Health, Fujita Health University, Aichi, Japan; 3 Department of Clinical Epidemiology, Translational Medical Center, National Center of Neurology and Psychiatry, Tokyo, Japan; 4 Department of Environmental Epidemiology, Institute of Industrial Ecological Sciences, University of Occupational and Environmental Health, Japan, Fukuoka, Japan; 5 Cancer Control Center, Osaka International Cancer Institute, Osaka, Japan

**Keywords:** work-related stress, pre-existing disease, job demand, job control, COVID-19

## Abstract

**Background:**

The job environment has changed a lot during the period of the coronavirus disease 2019 (COVID-19) pandemic. This study aimed to investigate the association between work-related stress and aggravation of pre-existing disease in workers during the first state of COVID-19 emergency in Japan.

**Methods:**

Data were obtained from a large internet survey conducted between August 25 and September 30, 2020 in Japan. Participants who reported that they had a job as well as current history of disease(s) (ie, pre-existing conditions) were included (*n* = 3,090). Aggravation of pre-existing disease during the state of emergency was self-reported. Work-related stress from April 2020 (since the state of COVID-19 emergency) was assessed according to a job demand–control model. Multivariable logistic regression models were used to analyze the association.

**Results:**

Aggravation of pre-existing diseases was reported by 334 participants (11%). The numbers of participants with high demand and low control were 112 (18%) and 100 (14%), respectively. Compared to medium demand, high demand was significantly associated with aggravation of pre-existing diseases (odds ratio 1.77; 95% confidence interval, 1.30–2.42). Low control compared to medium control was also significantly associated with aggravation of pre-existing diseases (odds ratio 1.39; 95% confidence interval, 1.02–1.92).

**Conclusion:**

Work-related stress during the first state of COVID-19 emergency was associated with aggravation of pre-existing disease during that period.

## INTRODUCTION

The World Health Organization (WHO) declared a global health emergency on January 30, 2020 due to coronavirus disease 2019 (COVID-19) caused by the severe acute respiratory syndrome coronavirus.^1^ Aiming to prevent the spread of the disease, countries worldwide implemented various degrees of restrictions in domestic socioeconomic activities, in addition to international quarantine.^2^

The Japanese government initiated the first state of COVID-19 emergency on April 7 in Tokyo, Osaka, Fukuoka, and nearby prefectures, which was later extended all across Japan from April 16. The restrictions were gradually relaxed and ended on May 25.^3^ During the emergency, citizens were urged to stay at home (stay-home order) but without legal enforcement. Many businesses, factories, and public facilities were closed following the order.

Several health-related issues have arisen directly or indirectly from the stay-home order. First, some healthcare facilities limited the number of patients, and some clinics were closed to avoid the spread of the infections during this period,^4^^,^^5^ which might have proved to be a disadvantage for people in general to seek health care services. Second, the work environment might have changed, resulting in an increase of work-related stress for some workers during the state of emergency.^6^^–^^8^ Third, essential workers had to continue working at the worksite, unlike many white-collar workers, who could work remotely.^9^ Some workers, depending on the employer, might have continued to work as the stay-home order was not mandatory in Japan.^10^^–^^12^

An international online survey reported on the rise in mental health issues and the related factors.^13^ However, characteristics of the work that were related to the worsening of health status, especially of the working population, have not been reported yet. Although working a high-strain job is reportedly related to the risk of cardiovascular diseases,^14^ whether it is associated with worsening of health status in a relatively short period of time is unknown. Therefore, we investigated the association between work-related stress and aggravation of pre-existing disease in workers with self-reported pre-existing health conditions in Japan.

## METHODS

### Study design and data sources

We used the data from the Japan COVID-19 and Society Internet Survey (JACSIS) study, which was conducted by Rakuten Insight, Inc., a large internet research agency.^15^ The JACSIS study used a web-based cross-sectional self-reported questionnaire survey for data collection. It covered 28,000 participants who were settled across all 47 prefectures in Japan. We inquired about histories of diseases; health care-seeking status; self-rated health status or aggravation; as well as work-related factors, including job stress. The survey also asked about lifestyles, such as drinking or smoking; family; and socio-economic status and changes. The study (JACSIS) attempted to illustrate health disparities with the aim of proposing evidence-based socio-economic relief and health promotion measures.^16^

### Participants

First, in an attempt to avoid including participants with the possibility of invalid responses, typically because of language obstacle (ie, providing responses without understanding the meaning of the survey items to receive compensation), a question item “Please select the penultimate one from the following options: 1)-A, 2)-B, 3)-C, 4)-D, 5)-E” was set. Those who did not select “4)” were excluded (*n* = 1,955), leaving 26,045 respondents eligible for the JACSIS study.

We included participants who had pre-existing disease identified using a question item “Do you have pre-existing disease (hypertension, diabetes, angina, myocardial infarction, stroke [cerebral infarction and cerebral hemorrhage], cancer, chronic obstructive pulmonary disease [COPD], asthma, bronchitis, pneumonia, atopic dermatitis, chronic pain lasting three months or more [eg, low back pain, headache], depression or other mental health disorders, periodontal disease, dental caries, and otitis media).” The possible responses for the question were: “1) No, 2) I used to have, but not anymore, 3) Yes, I have been under treatment, and 4) Yes, but I am not being treated.” Those who responded with “3)” or “4)” for at least one disease were defined as individuals with pre-existing disease (*n* = 11,783). Participants who have a job were identified using the question item “Please choose the response that matches your work situation (including on leave). If you have more than one job, please select one of the main jobs.” The possible responses included: “1) Executive of company (excluding self-employed); 2) Family- or self-operated business owner; 3) Member of family- or self-operated business; 4) Regular employee and working in a management position; 5) Regular employee and working not in a management position; 6) Dispatched employee; 7) Contract or temporary employee; 8) Part-time worker; 9) Home pieceworker; 10) Student, including one who is studying to retry the university entrance exam; 11) Retired; 12) Housewife/househusband; and 13) Unemployed.” Those who identified themselves as any one of “1)” through “9)” were defined as having a job (*n* = 15,838). Individuals who met both the criteria were included (*n* = 6,735).

As stated later, having aggravation of pre-existing disease was also inquired using a question item with three possible responses: “yes, no, and not applicable.” In the present analysis, participants who responded with “not applicable” (*n* = 3,638) were excluded. Additionally, seven participants who did not report their education completion year were excluded. The final sample size was 3,090 (aged 15–79) consisting of 2,002 men and 1,088 women.

### Variables

#### Outcome variable

Participants having aggravation of pre-existing disease were defined as those who responded “yes” for the question item “During the period of April to May in 2020 (state of the COVID-19 emergency), did you experience having aggravation of pre-existing disease?” Those who responded “no” were treated as the reference group.

#### Exposure variables

Work-related stress since the first state of COVID-19 emergency (from April 2020) was demonstrated according to the job demand–control model. This model is a well-known theory that explains how job characteristics influence employees’ psychological well-being.^17^ This model postulates that psychological strain results from the combined effects of the demands of the work situation and the range of decision-making freedom (control) available to the worker facing those demands.^17^ Job strain occurs when job demand is high and job control is low.^17^ In Japan, the New Brief Job Stress Questionnaire^18^ was modified based on the job demand–control model applicable to Japan’s occupational condition. It is implemented by the Japanese Ministry of Health, Labour and Welfare as a tool for measuring job strain.^19^ Items from the New Brief Job Stress Questionnaire were also included in JACSIS survey. In this study, work-related stress was evaluated based on those items. Demand and control were each divided into three groups (low, medium, and high) based on the standard cut-off points,^19^ where the medium group was treated as the reference. Job strain was defined as higher demand and lower control, where demand and control were dichotomized based on national standard mean of each.^20^ Lower demand and higher control were treated as reference. Details of the criteria are presented in eTable 1. Cronbach’s alpha was calculated with the data in this study, and high degree of internal consistency reliability was found (job demand: α = 0.83; control: α = 0.80).

#### Covariates

Type of pre-existing diseases was treated as a covariate. Pre-existing disease was determined by presence of at least one of the following eight diseases or conditions: hypertension, diabetes, cardiovascular disease (angina, myocardial infarction, and stroke), respiratory diseases (COPD, asthma, bronchitis, pneumonia), atopic dermatitis, chronic pain, cancer, mental health disorder (depression and other mental health disorders), and other diseases (periodontal disease, dental caries, and otitis media). We selected these diseases and conditions from their prevalence in Japan, and also from our perspective that the state of emergency should have had varying degrees of influence on typical health conditions, which requires description and exploration. Since the JACSIS study was designed to collect a wide range of sociodemographic, lifestyle, and health-related information from individuals aged 15–79 years trying to recruit a nationally representative sample, other diseases and conditions prevalent in younger age-groups were also inquired.

Education was divided into two groups: college or higher, and high school or lower. Income change compared to the pre-COVID-19 period was evaluated by response to a question item “How has your current household income changed, assuming your previous household income was 100? For example, answer 50 if it is reduced by half and 200 if it is doubled.” A number less than 100 indicates decreased income. Type of job content was divided into three groups according to the question item “Please choose the one that is closest to your job content: 1) Mainly desk work (office work and work using computer), 2) Mainly talking with people (marketing and sales), 3) Mainly physical work (eg, work at production site, nursing care).”

Smoking status was assessed by the frequency of using different kinds of tobacco products separately, including cigarette and heated tobacco. A current smoker is one who uses at least one kind of tobacco product occasionally or more often. Alcohol drinking habit was evaluated as the average amount of intake per day expressed in the Japanese unit *gō*, where 1 *gō* is equivalent to 23 g of ethanol.

Fear of COVID-19 was evaluated using a question item “How scared are you of Novel Coronavirus? 1) Not at all (0%), 2) No (25%), 3) Neutral (50%), 4) Yes (75%), 5) Yes, very much (100%).” Those who rated the fear 75% or over (4 or 5) were defined as “being afraid of COVID-19”.

### Statistical analyses

Statistical analyses of the current study were performed using Python 3.7 programming language (Python Software Foundation, http://www.python.org). The computational environment was Jupyter Notebook (Project Jupyter, http://www.jupyter.org).^21^ Means and standard deviations (SDs) were presented for continuous variables. Categorical variables were presented as proportions. Differences in means across three categories of demand or control status were tested using analysis of covariance (ANCOVA).

Multivariable binary logistic regression models were used to analyze the association between job demand or control status and the outcome variable of having pre-existing disease aggravation adjusted for demand and control mutually (model 1); age and sex (model 2); education, income change, smoking status, alcohol drinking habit, fear of COVID-19, type of pre-existing disease, and type of job content (model 3). Adjusted odds ratios (ORs) and 95% confidence intervals (CIs) derived from the models were reported. To check whether the association was mediated by refraining from medical visit, we also did a mediation analysis to estimate the indirect effect of job strain on aggravation of pre-existing disease. Details of the method and results are presented in eMaterials 1. All statistical tests were two-sided, and *P* < 0.05 was considered statistically significant.

### Ethics approval

This study was approved by the Bioethics Review Committee of Osaka International Cancer Institute, Japan. All procedures performed in this study were in accordance with the Ethical Guidelines for Medical and Health Research Involving Human Subjects enforced by the Ministry of Health, Labour and Welfare, Government of Japan, and with the 1964 Helsinki Declaration and its later amendments.

## RESULTS

Compared to those in the medium group, participants with higher demand or lower control levels were significantly younger (Table 1). They were more likely to be afraid of COVID-19, have an aggravation of pre-existing disease, and have decreased income after considering their age. Similarly, participants with a higher level of both demand and control were more likely to be current or ex-smokers, have higher daily alcohol consumption, and have a spouse. Participants with lower demand were less likely to have higher education, while no significant association was found between job control and education level. Those with higher control were more likely to engage in deskwork, the proportion of which, however, did not differ significantly among demand groups. The proportions of subjects having respiratory disease, chronic pain, mental health disorders, and atopic dermatitis were significantly higher, while the proportion of subjects having hypertension was significantly lower in participants with higher demand level. The proportion of subjects having mental health disorders was significantly higher in participants with lower control.

**Table 1.** Characteristics of participants by demand and control levels

	Demand				Control			
	
	Low *N* = 1,555	Medium *N* = 992	High *N* = 543	*P*-value	Low *N* = 646	Medium *N* = 1,001	High *N* = 1,443	*P*-value
Age								
Years	56.3 (13.0)	49.6 (13.4)	45.8 (12.1)	<0.001	49.6 (12.8)	50.4 (13.6)	54.7 (13.7)	<0.001

Sex^a^								
Women	31%	42%	33%	<0.001	31%	43%	32%	<0.001

Having spouse^a^								
Yes	63%	63%	66%	0.35	61%	61%	66%	<0.001

Current smoker^a^								
Yes	24%	28%	30%	<0.01	24%	25%	29%	0.01

Alcohol drinking^a^								
*gō* per-day	1.3	1.3	1.5	0.04	1.3	1.3	1.4	0.04

Education, less than college^a^								
Yes	28%	28%	22%	<0.02	24%	28%	27%	0.23

Decreased income^a^								
Yes	30%	36%	37%	<0.01	36%	32%	32%	0.17

Afraid of COVID-19								
Yes	61%	67%	72%	<0.001	66%	65%	64%	0.27

Type of pre-existing diseases								
Hypertension^a^								
Yes	52%	52%	45%	<0.01	51%	49%	52%	0.39
Diabetes^a^								
Yes	22%	24%	19%	0.08	22%	23%	22%	0.97
Cardiovascular disease^a^								
Yes	12%	13%	12%	0.71	12%	13%	13%	0.69
Respiratory disease^a^								
Yes	31%	36%	38%	<0.01	31%	36%	34%	0.10
Chronic pain^a^								
Yes	31%	38%	40%	<0.001	35%	35%	35%	0.95
Cancer^a^								
Yes	11%	13%	9%	0.09	9%	13%	11%	0.02
Mental health disorder^a^								
Yes	25%	29%	34%	<0.001	33%	28%	26%	<0.01
Atopic dermatitis^a^								
Yes	20%	23%	25%	0.04	20%	23%	22%	0.28
Other disease^a^								
Yes	80%	80%	80%	0.99	78%	81%	81%	0.40

Aggravation of pre-existing disease^a^								
Yes	9%	10%	18%	<0.001	14%	10%	10%	<0.01

Deskwork^a^								
Yes	51%	49%	49%	0.48	38%	48%	57%	<0.001

Variables are presented as means (standard deviations) or percentages for continuous or categorical variables, respectively.

^a^Results are adjusted for age.

High demand was significantly associated with aggravation of pre-existing diseases (OR 1.77; 95% CI, 1.30–2.42) compared to medium demand, independent of the degree of control (Table 2). Participants with low demand were significantly less likely to experience aggravation of pre-existing diseases in the model adjusted for control, which was significantly attenuated after further adjustment for age, sex, and other confounding factors. In contrast, low control was significantly associated with experiencing aggravation of pre-existing diseases, independent of demand and other confounding factors (OR 1.39; 95% CI, 1.02–1.92). High control was not associated with aggravation of pre-existing diseases when demand was simultaneously included in the model. Stratified analyses by sex yielded essentially the same results (data not shown).

**Table 2.** Odds ratios (95% confidence intervals) of being aggravation of pre-existing diseases with demand and control

	Model 1		Model 2		Model 3	
		
	Odds ratio	95% CI	Odds ratio	95% CI	Odds ratio	95% CI
Demand						
Low	0.65	0.49–0.86	0.89	0.66–1.18	1.03	0.76–1.40
Medium	Ref		Ref		Ref	
High	2.11	1.57–2.82	1.96	1.45–2.64	1.77	1.30–2.42

Control						
Low	1.37	1.01–1.84	1.42	1.05–1.94	1.39	1.02–1.92
Medium	Ref		Ref		Ref	
High	0.84	0.64–1.10	0.99	0.75–1.31	0.97	0.72–1.30

CI, confidence interval.

Model 1: adjusted for demand and control mutually.

Model 2: adjusted for age and sex in addition to model 1.

Model 3: adjusted for current smoker (yes/no), alcohol drinking (*gō* per day, 1 *gō* = 180 mL), afraid of COVID-19 (yes/no), education less than college (yes/no), type of pre-existing diseases, job contents (deskwork: yes/no), and decreased income (yes/no) in addition to model 2.

**Figure 1.**
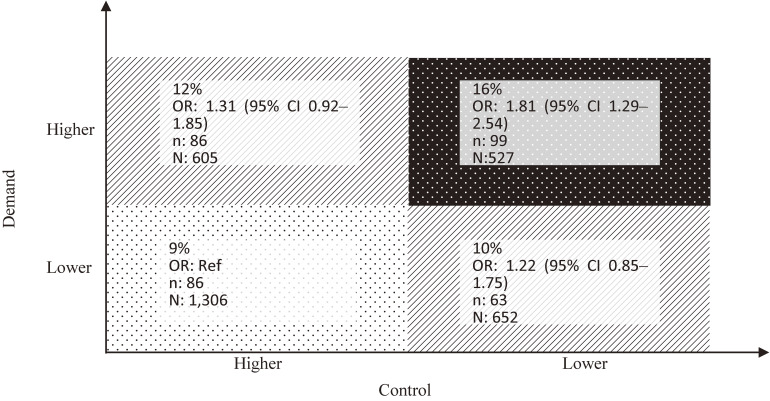
Percentages and odds ratios of participants whose pre-existing disease was aggravated according to job strain during the first state of COVID-19 emergency in Japan. Percentages (n/N) were adjusted for age and sex using general linear model. Odds ratios were calculated by multivariable logistic regression adjusted for age (continuous), sex, current smoker (yes/no), alcohol drinking (*gō* pre-day, 1 *gō* = 180 mL), afraid of COVID-19 (yes/no), education less than college (yes/no), type of pre-existing diseases, job contents (deskwork: yes/no), and decreased income (yes/no). CI, confidence interval; COVID-19, coronavirus disease 2019; OR, odds ratio; n, number of participants who had aggravation of the pre-existing diseases; N, number of participants in the respective category.

The prevalence of job strain was 17.1% (527/3,090), and the age-adjusted percentage of pre-existing disease aggravation in those with job strain was 16.0% (99/527), which was significantly greater than the reference group (low demand and high control) (age-adjusted percentage, 9% [86/1,306]; OR 1.81; 95% CI, 1.29–2.54, Figure 1). Job strain was not significantly associated with refraining from medical visits. Mediation analyses revealed that the indirect effect of job strain on aggravation of pre-existing disease through refraining from medical visits was not statistically significant (methods and results are presented in the eMaterials 1, eFigure 1, and eTable 1).

## DISCUSSION

This is the first study investigating the relationship between work-related stress and aggravation of pre-existing disease during the first state of COVID-19 emergency in Japan. We found that job strain was significantly associated with worsening of pre-existing disease. We considered the possible explanations for the association.

Although we did not inquire about the participants’ job strain before April 2020, there might have been changes in job strain during the first state of COVID-19 emergency. First, many companies experienced a decline in business performance. Many small- and medium-sized enterprises faced bankruptcy and layoffs, and some large companies chose to reduce their scale and adjust their operations,^22^ even though the Japanese government provided relief funds to individuals and companies.^23^ These might have intensified employees’ sense of occupational crisis. Second, there might have been workers who had to take on more workload, which may be due to structural adjustment of companies, such as reducing the number of employees.^24^ Furthermore, some might have experienced increased workload in the form of working from home, partly due to problems of network connection, communication among colleagues, environmental adaptation,^6^^,^^7^ and work–family conflicts.^25^

The present finding that job strain was associated with aggravation of pre-existing diseases is consistent with that of previous studies. For example, it has been reported that chronic job strain after a first myocardial infarction was associated with an increased risk of recurrent coronary heart disease in Canada and other countries.^26^^–^^28^ Work stress was reportedly associated with poor oral health condition.^29^^,^^30^ Also, the Employee Cohort Study in the COVID-19 Pandemic in Japan (E-COCO-J), which has been conducted online since March 2020 and surveying about 1,500 workers, reported that mental health had worsened among workers during the COVID-19 pandemic.^31^

The strength of this study lies in its large size and nationwide sampling design. However, this study is not without limitations. First, all the items were self-reported at the same time. Therefore, in addition to an issue of the validity, a significant limitation of the present study is that both the exposure variable (work-related stress from April 2020) and the outcome variable (aggravation of pre-existing disease during the period of April to May in 2020) were measured at the same time, and those who perceived their health status worsening (aggravation of pre-existing diseases) might have responded that they had high work-related stress (recall bias). Ideally, prospective studies are essential to overcome this limitation. Since the New Brief Job Stress Questionnaire has been conducted annually in Japan, retrospective cohort study of nested case-control studies should be conducted. Also, aggravation of pre-existing disease should also be assessed using different, more objective methods. Second, we only examined the association of job strain with the aggravation of pre-existing disease in a situation where the impact of COVID-19 on any of the variables could have existed. So it would not be possible for us to infer the influence of COVID-19 on the association between job strain and the aggravation of pre-existing disease. In an attempt to address whether those with job strain might be more susceptible to changes in the health status related to COVID-19 pandemic, we examined the association between job strain and the presence of symptoms. Participants with job strain were more likely to have symptoms that appeared after COVID-19 pandemic (16% vs 14%), as well as those that persisted from pre-COVID-19 (77% vs 69%). These results may indicate that participants with job strain might suffer poor health more than those without job strain, especially after COVID-19. Third, the results of the present study may not be generalizable to the general Japanese population due to its online survey design. Nevertheless, the JACSIS study implemented a simple random sampling procedure in strata defined by age, sex, and residential prefecture in an attempt to be representative of the Japanese population in these aspects. Finally, this is a cross-sectional study, so no causation can be implied. This study describes only associations, and further studies are needed to explore causality.

### Conclusions

This study found that work-related stress was associated with aggravation of pre-existing disease during the first state of COVID-19 emergency. These findings have important implications for socio-economics and public health in the work environment, especially regarding workers’ health.

## APPENDIX A. SUPPLEMENTARY DATA

eMaterials 1. Mediation analysis

eFigure 1. Path analysis

eTable 1. New Brief Job Stress Questionnaire: the English version^1^
