# Supplementary material for: The Association of Work-related Stress According to the Demand–Control Model With Aggravation of Pre-existing Disease During the First State of COVID-19 Emergency in Japan
Source: J Epidemiol. 2021 Dec 5;31(12):642–7. doi: 10.2188/jea.JE20210146 (PMC8593583; doi:10.2188/jea.JE20210146)
Supplement: Supplementary file 1 [file je-31-642-s001.pdf]

eMaterials 1. Mediation analysis

To examine whether the association between job strain and aggravation of pre-existing disease was mediated by refrained from medical visit, we also attempted a mediation analysis to estimate the indirect effect of job strain on aggravation of pre-existing disease.

Being refrained from medical visit or treatment was defined when participants reported "yes" at least once for the question item "During the period of April to May in 2020 (state of the COVID-19 emergency), did you have a time(s): 1) Could not go or refrained from going to hospital as scheduled; 2) Could not go or refrained from going to the hospital or receive an examination due to unexpected symptoms or medical conditions; 3) Could not be admitted or postponed the hospitalization; 4) Could not get a surgical procedure or postponed it; 5) Could not get non-surgical procedure or treatment or postponed it; 6) Could not go or refrained from going to dental clinic."

Standard path-analytic approaches[1, 2] were followed to assess: 1) the effect of job strain (X) on aggravation of pre-existing disease (Y) relative to "non-job strain" — *c* path; 2) the effect of job strain on proposed mediator (M), refraining from medical visit — *a* path; 3) the association between M and Y while statistically equating the effect of X on Y in the model — *b* path (**eFigure 1**).

Coefficients for *a* path were estimated using ordinary least-squares regression, whereas logistic regression was used for the coefficients of *b* and *c* paths. All of these analyses were adjusted for the covariates of age, sex, current smoker (yes/no), alcohol drinking (gō pre-day, 1 gō = 180 mL), afraid for COVID-19 (yes/no), education less than college (yes/no), type of pre-existing diseases, job contents (deskwork: yes/no), refrained from medical visit or treatment (yes/no), and decreased income (yes/no). Coefficients (β)

with 95% confidence intervals (CIs) were reported.

The *c* path represented the total effect of X on Y relative to the reference unadjusted for M. The *c′* path represented the direct effects of X on Y relative to the reference adjusted for M. The indirect effect through M of X relative to the reference on Y was represented by the products of coefficients from *a* and *b* path – *ab*. 95% CI for the indirect effect was generated from 10,000 bootstrap samples. Statistical significance is indicated when the CI values do not cross zero. Boot strapping is recommended for testing of indirect effects because it does not assume normality in sampling distribution.[2]

Job strain was not significantly associated with refraining from medical visit (β= 0.035; 95%CI, -0.014 to 0.083). Refraining from medical visit was significantly associated with aggravation of pre-existing disease (β=1.017; 95% CI, 0.759–1.275). However, the indirect effect of job strain on aggravation of pre-existing disease through refraining from medical visit was not statistically significant (β=0.021; 95% CI, -0.011 to 0.053) (**eFigure 1**).

REFERENCES

1. Hayes AF. Introduction to mediation, moderation, and conditional process analysis: A regression-based approach. New York: Guilford Press; 2013.

2. Hayes AF, Preacher KJ. Statistical mediation analysis with a multicategorical independent variable. Br J Math Stat Psychol. 2014;67(3):451-70.

eFigure 1. Path analysis

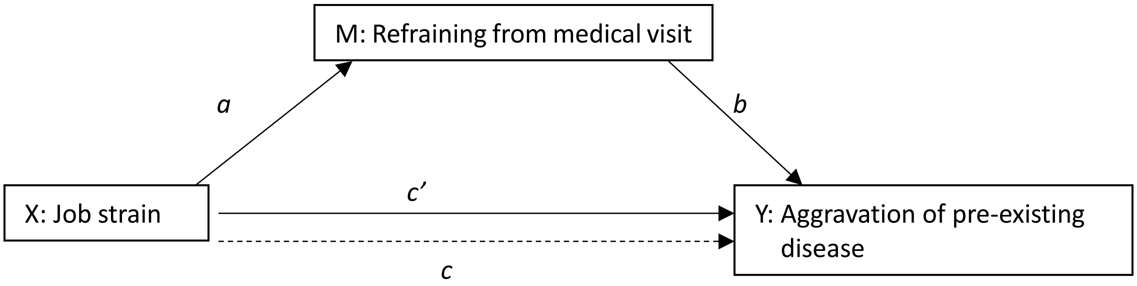

$M = a X + e_1$
$Y = c' X + b M + e_2$
$Y = c X + e_3$

c′: direct effect of X
c: total effect of X
ab: indirect effect of X

a = 0.035 (-0.014 to 0.083)
b = 1.017 (0.759 to 1.275)
c′ = 0.585 (0.243 to 0.926)
c = 0.594 (0.256 to 0.931)
ab = 0.021 (-0.011 to 0.053)

eTable 1. New Brief Job Stress Questionnaire: the English version[1]

	Very much so	Moderately so	Some what	Not at all
Demand				
1. I have an extremely large amount of work to do	4	3	2	1
2. I can't complete work in the required time	4	3	2	1
3. I have to work as hard as I can	4	3	2	1
Control				
1. I can work at my own pace	4	3	2	1
2. I can choose how and in what order to do my work	4	3	2	1
3. I can reflect my opinions on workplace policy	4	3	2	1

Demand and control are quantified by the sum of their three sub-items' scores, respectively. Cut-off for defining levels of demand and control: men: low demand 3–7, medium 8–9, high demand 10–12; women: low demand 3–6, medium 7–9, high demand 10–12. Men: low control 3–6, medium 7–8, high control 9–12; women: low control 3–5, medium 6–8, high control 9–12.[2]

Job strain was defined by higher demand and lower control, where demand and control

were dichotomized based on national standard mean of each: men: lower demand ≤8.7, higher demand >8.7, lower control ≤7.9, higher control >7.9; women: lower demand ≤7.9, higher demand >7.9, lower control ≤7.2, higher control >7.2.[3]

Original Japanese version

職業性ストレス簡易調査票の簡略化版

	そうだ	まあそうだ	ややちがう	ちがう
仕事の負担量				
非常にたくさんの仕事をしなければならない	4	3	2	1
時間内に仕事が処理しきれない	4	3	2	1
一生懸命働かなければならない	4	3	2	1
仕事のコントロール度				
自分のペースで仕事ができる	4	3	2	1
自分で仕事の順番・やり方を決める	4	3	2	1

ことができる				
職場の仕事の方針に自分の意見を反映できる	4	3	2	1

REFERENCES

1. Inoue A, Kawakami N, Shimomitsu T, et al. Development of a short questionnaire to measure an extended set of job demands, job resources, and positive health outcomes: the new brief job stress questionnaire. Ind Health. 2014;52:175-89.
2. Japan Ministry of Health, Labour and Welfare. An implementation manual for stress check system based on the Industrial Safety and Health Law [in Japanese]. https://www.mhlw.go.jp/content/000533925.pdf; Accessed 07.07.2021.
3. Kawakami N. Research on prevention of mental health problems of workers and improvement for work environment by stress check system: 2015-2017 comprehensive research report [in Japanese]. https://mental.m.u-tokyo.ac.jp/jstress/H27_29%E3%82%B9%E3%83%88%E3%83%AC%E3%82%B9%E3%83%81%E3%82%A7%E3%83%83%E3%82%AF%E7%8F%AD%E7%B7%8F%E5%90%88%E5%A0%B1%E5%91%8A%E6%9B%B8.pdf; Accessed 07.07.2021.
